# Propofol inhibits lung cancer cell viability and induces cell apoptosis by upregulating microRNA-486 expression

**DOI:** 10.1590/1414-431X20165794

**Published:** 2017-01-05

**Authors:** N. Yang, Y. Liang, P. Yang, T. Yang, L. Jiang

**Affiliations:** 1 Department of Anesthesiology, The First Affiliated Hospital of Wenzhou Medical University, Wenzhou, Zhejiang Province, China; 2 Department of Pediatric Intensive Care Unit, The Second Affiliated Hospital of Wenzhou Medical University, Wenzhou, Zhejiang Province, China

**Keywords:** Propofol, Lung cancer, Cell viability, Cell apoptosis, microRNA-486

## Abstract

Propofol is a frequently used intravenous anesthetic agent. Recent studies show that propofol exerts a number of non-anesthetic effects. The present study aimed to investigate the effects of propofol on lung cancer cell lines H1299 and H1792 and functional role of microRNA (miR)-486 in these effects. H1299 and/or H1792 cells were treated with or without propofol and transfected or not with miR-486 inhibitor, and then cell viability and apoptosis were analyzed by 3-(4,5-dimethylthiazol-2-yl)-2,5-diphenyltetrazolium bromide (MTT) and flow cytometry. The expression of miR-486 was determined by quantitative real-time polymerase chain reaction (qRT-PCR) with or without propofol treatment. Western blot was performed to analyze the protein expression of Forkhead box, class O (FOXO) 1 and 3, Bcl-2 interacting mediator of cell death (Bim), and pro- and activated caspases-3. Results showed that propofol significantly increased the miR-486 levels in both H1299 and H1792 cells compared to untreated cells in a dose-dependent manner (P<0.05 or P<0.01). Propofol statistically decreased cell viability but increased the percentages of apoptotic cells and protein expressions of FOXO1, FOXO3, Bim, and pro- and activated caspases-3; however, miR-486 inhibitor reversed the effects of propofol on cell viability, apoptosis, and protein expression (P<0.05 or P<0.01). In conclusion, propofol might be an ideal anesthetic for lung cancer surgery by effectively inhibiting lung cancer cell viability and inducing cell apoptosis. Modulation of miR-486 might contribute to the anti-tumor activity of propofol.

## Introduction

Lung cancer is the leading cause of cancer-related death in both men and women worldwide (1), accounting for approximately 28% of all cancer deaths (2). In 2012, more than 1.8 million newly diagnosed lung cancer cases and about 1.6 million deaths were estimated (3). It has been reported that more than 1/3 of all new lung cancer cases were diagnosed in China, leading to a major burden for both patients and their families (4). Moreover, the incidence of lung cancer has been reported to be increasing in China (4). Although tremendous advance has been made in recent years in prevention and treatment of lung cancer, the mortality is still very high because of the unclear molecular pathogenesis, with a 5-year survival rate of less than 16% for patients with advanced stage (5).

MicroRNAs (miRNAs) are a class of endogenous, single-stranded, small, non-coding RNAs that negatively regulate gene expression at the post-transcriptional level by mainly binding to 3′-untranslated region (UTR) of target mRNAs, leading to mRNA degradation or translational inhibition (6). It has been well acknowledged that miRNAs play an essential role in various biological processes such as cell proliferation, apoptosis, differentiation, migration and invasion (7). An increasing number of studies have suggested that miRNAs are involved in the pathophysiology of cancers, and may provide a new insight into cancer treatment (8,9). Aberrant expression of miRNAs have been well described in lung cancer (10,11). Among miRNAs, miR-486 has been confirmed as a novel tumor-suppressor miRNA in many cancers including lung cancer (12,13). Therefore, upregulation of miR-486 expression might be a potential treatment for lung cancer.

Propofol is a frequently used intravenous anesthetic agent. Accumulating evidence suggests that propofol has a number of non-anesthetic effects (14). Recently, propofol was reported to show many potential anticancer properties, such as inhibition of proliferation, adhesion, and metastasis of cancer cells and induction of cell apoptosis (15–17). Hence, propofol is considered a better anesthetic agent than other anesthetics during cancer surgery (18). However, little information is available about the antitumor activity of propofol in lung cancer cells. Therefore, the present study aimed to explore the effects of propofol on the biological behavior of lung cancer cells, as well as the related underlying molecular mechanisms.

## Material and Methods

### Cell culture

Lung cancer cell lines H1299 and H1792 were obtained from the American Type Culture Collection (ATCC, USA). The cells were cultured in Roswell Parker Memorial Institute (RPMI)-1640 medium (Sigma-Aldrich, USA) supplemented with 10% fetal bovine serum (FBS, Gibco, USA) 100 U/mL penicillin (Gbico), and 100 mg/L streptomycin (Gbico) at 37°C in a humidified atmosphere with 5% CO_2_.

### Cell treatment and transfection

For cell treatment, cells were incubated with different concentrations of propofol (0, 5, and 10 μM) for 24 h. For cell transfection, miR-486 inhibitor was designed and produced by GenePharma (China). Cells without any vector transfection were considered the control group. Briefly, the cells (2×10^4^ cells/per well) were seeded on 96-well plates and incubated with or without propofol. Subsequently, the cells were then transiently transfected with miR-486 inhibitor according to the manufacturer’s manual by using lipofectamine 2000 (Invitrogen, USA) for 48 h. The cell suspension was collected for further analyses.

### Quantitative real-time polymerase chain reaction (qRT-PCR) analysis

After 24 h of incubation with different concentrations of propofol, the cells were collected and washed with PBS. Total RNA was extracted from the cells using TRIzol reagent (Invitrogen) according to the manufacturer’s protocol. Total RNA was reverse-transcribed and amplified with the Taqman MicroRNA Reverse Transcription kit (Applied Biosystems, USA) following the manufacturer’s instructions. PCR reactions were carried out with Taqman 29 Universal PCR master mix (Applied Biosystems), with U6 snRNA as a loading control. The reverse transcription and primers were both acquired from Genepharma. All reactions were performed in triplicate and the data were analyzed by the ddCt method.

### Cell viability

Cell viability was analyzed by 3-(4,5-dimethylthiazol-2-yl)-2,5- diphenyltetrazolium bromide (MTT) colorimetric assay according to a standardized method (19). Briefly, the cells were inoculated into 96-well plates, treated with propofol and transfected or not with miR-486 inhibitor. Forty-eight hours later, 20 μL 5 mg/mL MTT (Gibco BRL-Life Technologies, USA) was added to the plates and incubated at 37°C for 4 h. The cells were then added with dimethylsulfoxide (DMSO; 100 μL; Sigma-Aldrich) to dissolve the formazan crystals. Absorbance was read at 590 nm, determined with a microplate reader (Bio-Rad Benchmark, USA).

### Apoptosis assay

Cell apoptosis was determined by an Annexin V/FITC and propidium iodide (PI) apoptosis detection kit (Becton Dickinson, USA) according to the manufacturer's instructions. Briefly, 48 h after treatment and transfection, cells were collected and suspended in Annexin-binding buffer. The cells were then incubated with Annexin V-FITC and PI for 15 min in the dark at room temperature. Flow cytometry was performed to analyze the apoptotic percentage of cells.

### Western blot analysis

After incubation and transfection, the cell suspension was harvested, centrifuged, and lysed in a lysis buffer. Total protein was extracted from the cells and measured using a BCA assay kit (Pierce, USA). After boiling for 10 min, the protein samples were subjected to a 10–12% sodium dodecyl sulfate (SDS)-polyacrylamide gel electrophoresis (PAGE) and transferred to polyvinylidene fluoride (PVDF) membranes (Bio-Rad Benchmark). Subsequently, the cells were washed with phosphate buffer saline (PBS) and blocked with 5% skim milk in Tris buffered saline with tween (TBS-T) for 1 h. The membranes were then maintained in the following primary antibodies overnight at 4°C: anti-FOXO1 antibody (ab52857, Abcam, UK), anti-FOXO3 antibody (ab47285, Abcam), anti-Bcl-2 interacting mediator of cell death (Bim) antibody (ab32158, Abcam), anti-pro-caspase-3 antibody (ab32150, Abcam), and anti-active caspase-3 antibody (ab2302, Abcam). Membranes were washed twice with TBS-T and then incubated with horseradish peroxidase-conjugated secondary antibodies for 2 h. The protein bands were visualized with WEST-ZOL-plus Western Blot Detection System (Intron Biotechnology, Inc., Korea). Probing for GAPDH was used as a loading control.

### Statistical analysis

All samples were run in triplicate. Data are reported as means±SD. GraphPad Prism 6 software (GraphPad, USA) was employed for statistical analyses. Statistical differences were assessed by paired *t*-tests or one-way analysis of variance (ANOVA). P*<*0.05 was defined as statistically significant.

## Results

### Propofol stimulated miR-486 expression

**Figure 1:**
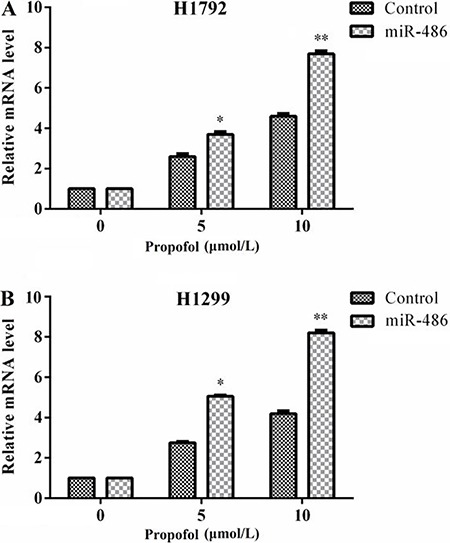
Lung cancer cell lines were treated or not with propofol for 24 h, and the expression of miR-486 was determined by qRT-PCR. Propofol significantly increased the levels of miR-486 in H1792 (*A*) and in H1299 (*B*) cells. Data are reported as mean ±SD. MiR: microRNA; qRT-PCR: quantitative real-time polymerase chain reaction. *P<0.05 compared to the control group; **P<0.01 compared to the control group (*t*-test).

As shown in Figure 1A, treatment with 5 or 10 μM propofol significantly increased the levels of miR-486 in H1792 cells compared to the untreated cells (P<0.05 or P<0.01), in a concentration-dependent manner. Thus, we chose 10 μM propofol for further analyzes. The expression pattern of miR-486 in H1299 cells showed similar results (Figure 1B). The results indicated that propofol could stimulate the expression of miR-486.

### Suppression of miR-486 reversed cell viability reduced by propofol in both H1299 and H1792 cells

**Figure 2:**
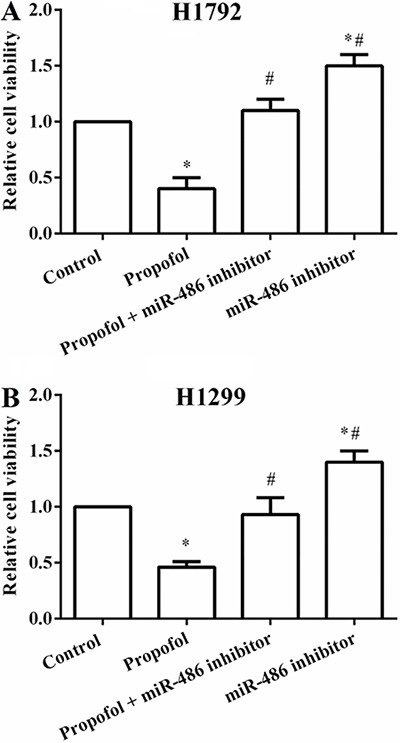
Relative cell viability in the different groups. Cell viability was significantly decreased by propofol, but these effects were reversed by transfection with miR-486 inhibitor in H1792 (*A*) and in H1299 (*B*) cells. Data are reported as mean ±SD. MiR: microRNA. *P<0.05 compared to the control group; ^#^P<0.05 compared to the propofol group (*t*-test).

Lung cancer cell lines H1792 and H1299 cells were treated with or without 10 μM propofol and transfected or not with miR-486 inhibitor. MTT was performed to determine the cell viability. The results showed that the cell viability in both H1792 and H1299 cells was significantly decreased by propofol compared to the control groups (P<0.05). However, cell viability was significantly elevated by propofol combined with miR-486 inhibitor compared to the propofol group (P<0.05). Further, the cell viability was dramatically higher by transfection with miR-486 compared to the propofol group or control group (P<0.05; Figure 2A and B). The results suggested that miR-486 suppression reversed the cell viability reduced by propofol.

#### Suppression of miR-486 reversed cell apoptosis increased by propofol in H1299 cells

**Figure 3:**
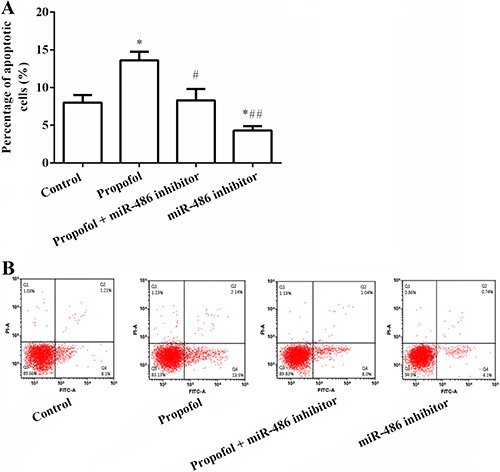
*A*, Percentages of apoptotic cells in the different groups measured by Annexin V/FITC and PI staining. Apoptotic cells were significantly increased by propofol, but these effects were reversed by transfection with miR-486 inhibitor in H1299 cells. *B*, Flow cytometry of cell apoptosis. Data are reported as mean ±SD. MiR: microRNA; PI: propidium iodide. *P<0.05 compared to the control group; ^#^P<0.05 compared to the propofol group; ^# #^P<0.01 compared to the propofol group (*t*-test).

As indicated in Figure 3A and B, the percentages of apoptotic cells were significantly upregulated by administration of propofol compared to the control group (P<0.05) in H1299 cells. But the percentages of apoptotic cells were significantly downregulated by propofol combined with miR-486 inhibitor compared to the propofol group (P<0.05), whereas, the percentages of apoptotic cells were markedly increased by miR-486 inhibitor compared to the propofol group or control group (P<0.05 or P<0.01). The results demonstrated that miR-486 suppression reversed the cell apoptosis increased by propofol.

#### Propofol regulated cell viability and apoptosis by regulating miR-486/FOXO in H1299 cells

**Figure 4:**
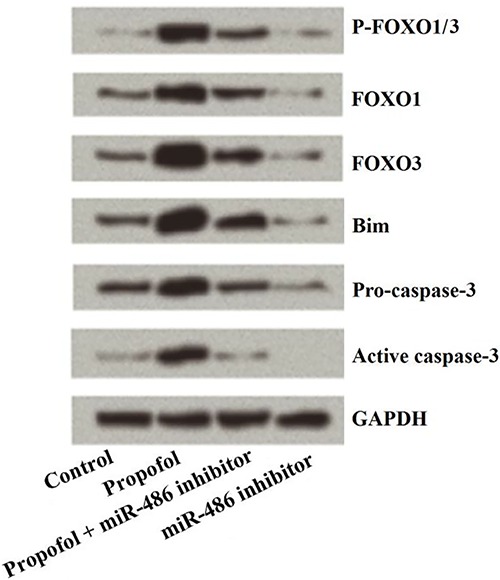
Western blot results for cell viability and apoptosis in H1299 cells, showing that the expressions of all evaluated proteins were significantly elevated by propofol, but these effects were reversed by transfection with miR-486 inhibitor in H1299 cells. MiR: microRNA; FOXO: forkhead box, class O; Bim: Bcl-2 interacting mediator of cell death.

As indicated in Figure 4, the expressions of FOXO1, FOXO3, Bim, pro- and activated caspase-3 were all significantly elevated by the administration of propofol compared to the control group in H1299 cells, but were significantly decreased by miR-486 inhibitor. Moreover, the effects of propofol on these proteins were alleviated by simultaneous treatment with propofol and transfection with miR-486 inhibitor. These data demonstrated that propofol regulated H1299 cell viability and apoptosis by regulating miR-486/FOXO.

### Discussion

Lung cancer is a major cause of mortality worldwide, and the clinical outcome is still poor in spite of early detection and treatment. Therefore, there is an urgent need to improve the current treatment or develop new therapeutic strategies. In the present study, we investigated the effects of propofol on the biological behavior of lung cancer cells and the role of miR-486 in these effects. The results revealed that propofol could inhibit lung cancer cell viability and promote cell apoptosis. Besides, we observed that propofol stimulated the expression of miR-486, and suppression of miR-486 reversed the effects of propofol on lung cancer cell viability, apoptosis, and expression of apoptosis-related protein. Hence, our results suggest that propofol might be an ideal anesthetic for lung cancer surgery.

Anesthesia is a reliable method for reducing pain and is widely used during surgery. Numerous anesthetics have been reported to be used during cancer resection; however, the effects of these anesthetics on the behavior of cancer cells are uncertain (20). Propofol is a safe and an effective alternative for sedation. Some advantages, such as rapid onset and short duration of action, have been reported for propofol. Therefore, intravenous anesthesia with propofol is growing in popularity. Nevertheless it is noteworthy that, different concentrations of propofol reveal contradictory results in different types of cancer cells. For example, Garib et al. (21) suggested that 34 μM propofol significantly elevated the migration of MDA-MB-468 breast carcinoma cells. Conversely, Mammoto et al. (22) observed that 5.6–28 μM propofol statistically reduced the invasion capacity of many human cancer cells including HeLa, HT1080, HOS and RPMI-7951. In addition, Miao et al. (23) demonstrated that 45 μM propofol markedly inhibited the invasion ability of colon carcinoma cells. Thus, in the present study, a concentration range of propofol (0–10 μM) was explored in respect to its effect on the behavior of lung cancer cell lines H1299 and H1792. In line with the above studies, our data also exhibited that administration of propofol inhibited cell viability and promoted cell apoptosis.

An increasing number of studies have revealed that propofol might regulate the biological behaviors of cancer cells by upregulating or downregulating the expression of miRNAs. For example, Su et al. (24) observed that propofol induced epithelial ovarian cancer cell apoptosis by upregulating the expression of miR-let-7i. Zhang et al. (25) suggested that propofol could effectively inhibit the adhesion of hepatocellular carcinoma (HCC) cells by upregulating miRNA-199a and downregulating metalloprotease-9 expression. Ye et al. (26) found that propofol inhibited the proliferation and invasion of osteosarcoma cells by the regulation of miR-143 expression. Wang et al. (17) demonstrated that propofol suppressed proliferation and invasion of pancreatic cancer cells by upregulating miR-133a expression. Similarly, in the present study we revealed that propofol inhibited lung cancer cell viability and induced cell apoptosis by upregulating miR-486 expression. To elucidate the underlying mechanism involved in the inhibition of H1299 and H1792 cells, the effects of propofol on the expression of miR-486 was determined. We found that propofol could distinctly increase the levels of miR-486. More importantly, suppression of miR-486 by transfection with miR-486 inhibitor reversed the effects of propofol on lung cancer cells viability and apoptosis. These results indicated that the anti-tumor function of propofol might be partly due to the upregulation of miR-486.

The functional role of miR-486 in cancers has been extensively investigated. It has been demonstrated that miR-486 functions as a tumor-suppressor miRNA in many cancers such as breast cancer (27), HCC (28), and gastric cancer (29). Recently, studies have shown that miR-486 was downregulated in lung tumors compared with adjacent uninvolved lung tissues and that miR-486 might play an important role in the development of lung cancer (12,13). In addition, miR-486 has been considered as a biomarker for early diagnosis and recurrence of non-small cell lung cancer (NSCLC) (30). To further explore the related mechanism underlying miR-486 suppression of cell viability and promotion of cell apoptosis, the expression of FOXO 1 and 3, Bim, and pro- and activated caspase-3 was analyzed. It has been reported that FOXO transcription factors have the ability of regulating various cell functions such as cell proliferation, apoptosis, cell survival, metabolic processes, and DNA repair (31–33). FOXO is involved in cell growth and apoptosis by directly inducing the expression of FOXO3a-dependent apoptotic protein Bim (34,35) and by activating caspase family (36). Moreover, FOXO stimulation could regulate specific gene expression, resulting in cell-cycle arrest, which implies that FOXO is responsible for the suppression of tumors (31). FOXO1 and FOXO3 (or FOXO3a) are two important members of FOXO family, which play critical roles in cell proliferation, cell cycle arrest and apoptosis (31,37). A previous study showed that the expression of FOXO1 was a favorable prognostic factor in NSCLC (38). In addition, inactivation of FOXO3a occurs frequently in carcinogen-induced lung adenocarcinoma (39) and FOXO3a could regulate the cytotoxic effects of cisplatin in lung cancer cells (40). As indicated in our results, administration of propofol significantly increased the protein levels of FOXO1, FOXO3, Bim, pro-caspase-3 and activated caspase-3, while transfection with miR-486 inhibitor showed contrary results. Suppression of miR-486 reversed the effects of propofol on the expression of apoptosis-related protein.

In conclusion, our results suggest that propofol could effectively inhibit lung cancer cell viability and induce cell apoptosis, and that propofol might be an ideal anesthetic for lung cancer surgery. Modulation of miR-486 might contribute to the anti-tumor activity of propofol.
